# SBRT combined with PD-1/PD-L1 inhibitors in NSCLC treatment: a focus on the mechanisms, advances, and future challenges

**DOI:** 10.1186/s13045-020-00940-z

**Published:** 2020-07-28

**Authors:** Yu Chen, Min Gao, Zhaoqin Huang, Jinming Yu, Xiangjiao Meng

**Affiliations:** 1 Cheeloo College of Medicine, Shandong University, Jinan, Shandong China; 2 Department of Radiation Oncology, Shandong Cancer Hospital and Institute, Shandong First Medical University and Shandong Academy of Medical Sciences, Jinan, Shandong China; 3 Department of Radiology, Shandong Provincial Hospital, Shandong First Medical University, Jinan, Shandong China

**Keywords:** Stereotactic body radiation therapy (SBRT), PD-1/PD-L1 inhibitors, Combination treatment, Non-small-cell lung cancer (NSCLC), Advances, Challenges

## Abstract

Immune checkpoint inhibitors targeting programmed cell death 1 (PD-1), programmed cell death ligand-1 (PD-L1), and others have shown potent clinical efficacy and have revolutionized the treatment protocols of a broad spectrum of tumor types, especially non–small-cell lung cancer (NSCLC). Despite the substantial optimism of treatment with PD-1/PD-L1 inhibitors, there is still a large proportion of patients with advanced NSCLC who are resistant to the inhibitors. Preclinical and clinical trials have demonstrated that radiotherapy can induce a systemic antitumor immune response and have a great potential to sensitize refractory “cold” tumors to immunotherapy. Stereotactic body radiation therapy (SBRT), as a novel radiotherapy modality that delivers higher doses to smaller target lesions, has shown favorable antitumor effects with significantly improved local and distant control as well as better survival benefits in various solid tumors. Notably, research has revealed that SBRT is superior to conventional radiotherapy, possibly because of its more powerful immune activation effects. Thus, PD-1/PD-L1 inhibitors combined with SBRT instead of conventional radiotherapy might be more promising to fight against NSCLC, further achieving more favorable survival outcomes. In this review, we focus on the underlying mechanisms and recent advances of SBRT combined with PD-1/PD-L1 inhibitors with an emphasis on some future challenges and directions that warrant further investigation.

## Background

Currently, NSCLC, which accounts for 80–85% of all lung cancer cases, remains one of the most malignant tumors worldwide and is a leading cause of cancer-related mortality [1]. In contrast to the advances in treatment for most tumors that have significantly increased survival, progression in NSCLC treatment has been slow, with a 5-year survival rate of 19% for all stages [2]. Continued exploration has been conducted to attain effective treatment protocols.

With the rapid development of immunotherapy, immune checkpoint inhibitors targeting PD-1/PD-L1 have exhibited encouraging therapeutic effects. At present, PD-1/PD-L1 inhibitors, including pembrolizumab and atezolizumab, have been recommended as first-line treatments for advanced NSCLC patients with high PD-L1 expression [3–7]. Compared with conventional chemotherapy, substantially improved survival has been demonstrated in second-line treatment [8–11]. Despite impressive achievements, limitations exist with only a small proportion of patients benefiting from PD-1/PD-L1 inhibitors [12]. In addition, acquired resistance with unclear mechanisms might eventually develop. To overcome resistance, a great deal of exploration has been done, with combination treatment becoming the most promising protocol.

Radiotherapy is a conventional treatment that is part of the standard of curative or palliative care for the majority of cancers. Evidence has revealed that radiation can exert potent immunomodulatory effects, potentially providing a supportive immune microenvironment for antitumor immunity [13, 14]. SBRT, also known as stereotactic ablative radiotherapy (SABR), is a novel radiotherapy modality with growing evidence indicating its significant efficacy in various solid tumors, especially early stage and oligometastatic NSCLC [15–17]. It can deliver high doses to relatively small target lesions, thus achieving more than 90% local control and substantially improving prognosis with a low risk of toxicity [18]. Of note, SBRT has been demonstrated to have remarkable advantages over conventional radiotherapy, potentially due to its more potent immune activation effects [19, 20]. Several studies have observed that radiation-induced antitumor immunity might be dose-dependent, with relatively higher doses being more powerful immunologic adjuvants [21, 22]. The superiority of SBRT to conventional radiotherapy makes it more favorable to combine with PD-1/PD-L1 inhibitors to achieve better survival benefits. To date, several preclinical and clinical trials have been conducted to explore whether this combination treatment has significant synergistic effects, with several more trials still undergoing.

Herein, we review the combination of SBRT with PD-1/PD-L1 inhibitors for the treatment of NSCLC, with some focus on the underlying mechanisms and recent advances. In addition, we also pose some future directions and challenges that warrant further investigation.

## The potential mechanisms of SBRT and PD-1/PD-L1 inhibitors in antitumor immunity

### The role of SBRT in immunomodulation

**Fig. 1:**
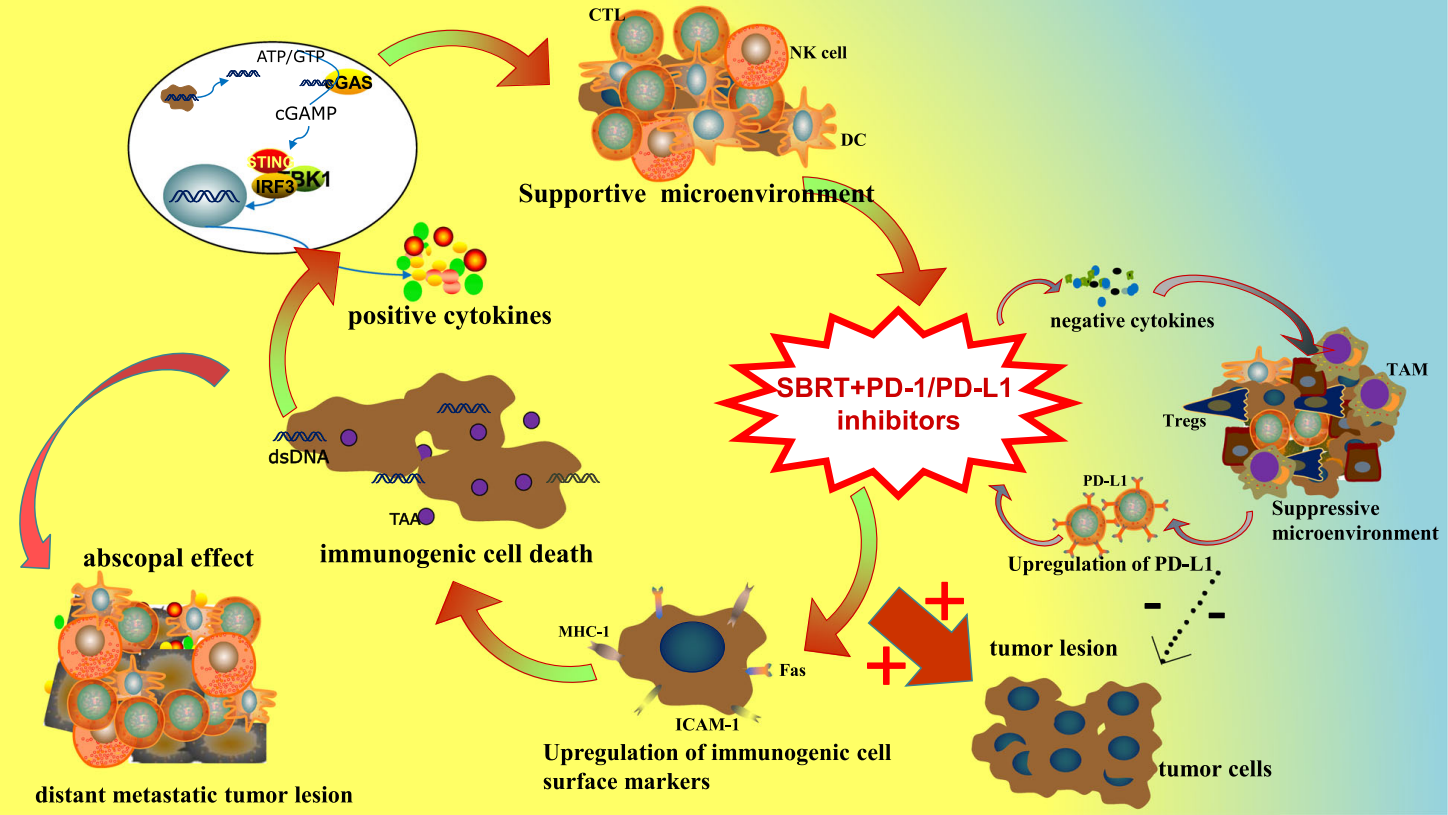
The specific mechanisms of SBRT combined with PD-1/PD-L1 inhibitors. The supportive modulatory mechanisms include upregulation of immunogenic cell surface markers such as ICAM-1, MHC-1 and Fas, induction of immunogenic cell death, release of tumor antigen and cytokines as IFN, TNFα, IL-1, IL-6, and so on, and enhanced homing of immune cells to tumors. Notably, the activated immune response can further act on distant nonirradiated metastases to appreciably inhibit metastases progression. Besides, SBRT can also induce immunosuppressive effects involving increased release of negative cytokines like TGFβ, accumulation of radioresistant suppressor cells, and upregulation of PD-L1 expression. The integration of PD-1/PD-L1 inhibitors to SBRT could not only enhance positive immunoregulation, but also significantly attenuate negative immune resistance, thus achieving potent anti-tumor immunity. Challenges exist to eliminate the remained suppressive effects

The role of SBRT in immunomodulation has gained extensive attention. Current evidence suggests that SBRT is involved in a variety of immunomodulatory processes and plays a significant role in antitumor immunity (Fig. 1) [13].

Hypofractionated radiation can upregulate the expression level of immunogenic cell surface markers such as ICAM-1, MHC-1, and Fas in a dose-dependent manner between 1 and 20 Gy with higher doses driving more marker expression [22–24]. Typically, the downregulation of immunogenic cell surface markers was observed in various tumors, which is an important cause of immune resistance and immune escape. The upregulation of these markers by SBRT enables the immune system to respond swiftly to abnormal changes in cells, thus resulting in enhanced antitumor effects [25]. In addition, preclinical studies have demonstrated that relatively high dose radiation can significantly induce intracellular stress, especially DNA damage mediated by reactive oxygen species (ROS), leading to the occurrence of immunogenic cell death (ICD) [26]. Evidence has shown that ICD can promote the maturation and presentation of dendritic cells (DCs) as well as the cross-priming of cytotoxic T lymphocytes (CTLs), mainly mediated by three distinct molecules, calreticulin, high-mobility group box 1 protein (HMGB1), and ATP [26–28]. Moreover, research has found that hypofractionated SBRT (single fraction 20–24 Gy) can trigger the exposure and release of adequate amount of tumor associated antigens (TAAs), especially damaged double stranded DNA (dsDNA), via ICD, which are further ingested by DCs and mediate the transmission of immune activation signals [29–31]. Cytoplasmic dsDNA is sensed by the cyclic GMP-AMP synthase (cGAS)-stimulator of interferon genes (STING) pathway to stimulate the generation of IFN-I, a key mediator that bridges innate and adaptive immune responses [32–34]. In addition, an increased influx of CTLs and a decreased influx of Tregs have been found in mouse tumors when irradiated with a high single dose of 10 Gy [35]. It appears that hypofractionated radiation could boost immune cell recruitment to irradiated tumor tissue by facilitating the release of chemokines and altering the vascular phenotype [36, 37]. The enhanced homing of immune cells to tumors offers a more supportive immune microenvironment to exert an effective antitumor response.

Through its positive immunomodulatory effects, SBRT can not only activate innate immune signaling pathways but also potentially induce adaptive immune responses within tumors in the radiation field as well as in distant metastases, which is called the abscopal effect [14, 29]. Danger signals released by the effects of SBRT turn into a very efficient in situ vaccine, resulting in the priming of CTLs and the production of related cytokines, especially IFN-γ, which further act on distant nonirradiated metastases to appreciably inhibit metastatic tumor progression [38]. It has been found that the expression of IFN-γ-associated genes is significantly correlated with the distant non-irradiated tumor response [39]. Notably, compared with a single dose of 20 or 30 Gy, the regimen of 8 Gy delivered in 3 fractions, though it achieved comparable irradiated tumor control, led to increased IFN-I gene expression and had superior abscopal effects [31, 40].

Conversely, in addition to the positive immunomodulatory effect, SBRT can also induce an immunosuppressive microenvironment. Evidence indicates that hypofractionated radiation could result in a significant increase in transforming growth factor β (TGFβ) [13]. TGFβ can not only affect CD8+ T cell proliferation and function but also induce CD4+ T cells to adopt a regulatory phenotype (Treg), thus dampening the radiation-induced antitumor immune response. In addition, preclinical research has shown that the ablative radiation of a single dose of 12 Gy plays an unexpected role in the upregulation of PD-L1 expression, which is mainly dependent on IFN-γ produced by CD8+ T cells [41, 42]. Increased PD-L1 could induce enhanced immunosuppression by binding to its receptor PD-1, which in turn leads to high radiation resistance. It has been demonstrated that irradiated tumors showed enhanced tumor-associated macrophage (TAM) infiltration, possibly attributed to the upregulation of chemoattractant stromal cell-derived factor 1 (SDF-1) and colony-stimulating factor 1 (CSF-1) as well as C-X-C chemokine receptor type 4 [37, 43, 44]. Typically, increased TAMs could promote tumor growth, invasion, and metastasis by negatively regulating antitumor immunity, thus leading to worse tumor suppression [45]. Moreover, evidence has shown that SBRT with a single dose of 12 Gy or 15 Gy can give rise to the recruitment of CD4 + T cells mainly composed of Foxp3+ Tregs [25, 46]. Further, study indicated that the Tregs increase induced by radiation was dose-dependent with a single dose of 20 Gy doubling that of a single dose of 2 Gy [47]. The accumulated Tregs in the tumor microenvironment significantly abrogate antitumor immune responses, which confers surviving tumor cells with potent radioresistance. Furthermore, Vanpouille Box et al. demonstrated that a DNA exonuclease, Trex1, might be activated with radiation doses above 12 to 18 Gy in a single fraction, which leads to the degradation of cytosolic dsDNA and further downregulates the activation of the immune systemic response by hindering IFN-β production and CTLs priming [40].

### Targeting the PD-1/PD-L1 signaling pathway for immune activation

PD-1 is an inhibitory transmembrane receptor of the CD28 family that is mainly expressed in T cells, B cells, natural killer (NK) cells, and many other tumor-infiltrating lymphocytes [48]. PD-L1, as the PD-1 ligand, is a transmembrane protein of the B7 family that is mostly found on the surface of antigen-presenting cells (APCs) as well as tumor cells. It is well acknowledged that the interaction of PD-1 with PD-L1 could markedly suppress the antitumor immunity of CTLs, thus leading to immune escape and resistance [49, 50]. Immune checkpoint inhibitors targeting PD-1/PD-L1 could block the negative regulatory pathway, which further makes an effort to reinvigorate dampened T cells to exert potent immune responses [51]. Nonetheless, the low PD-L1 expression on tumor cells and the immunologically “cold” tumor microenvironment that lacks sufficient T lymphocytes cause patients to be less responsive to PD-1/PD-L1 inhibitors [52, 53].

### The combination of SBRT and PD-1/PD-L1 inhibitors optimizes antitumor immunity

The immunomodulatory mechanisms of SBRT and PD-1/PD-L1 inhibitors in antitumor immunity shed light on the potential synergistic effect of the two in tumor treatment. The combination of SBRT and PD-1/PD-L1 inhibitors could not only enhance positive immunoregulation but also significantly attenuate negative immune resistance, thus garnering substantial promise for superior survival prognosis (Fig. 1).

SBRT can provide a more supportive immune microenvironment for subsequent PD-1/PD-L1 inhibitor treatment. It has been demonstrated that SBRT can promote the release of TAAs, which further induces DC maturation, the cross-priming of CTLs, and lymphocyte recruitment to tumors, thus converting immunologically “cold” tumors to “hot” tumors [54]. Therefore, poorly immunogenic tumors can overcome immune escape and immune resistance to PD-1/PD-L1 inhibitors by the priming effects of SBRT [55]. In addition, it is generally acknowledged that PD-L1 expression is one of the most representative predictive biomarkers of PD-1/PD-L1 inhibitors. The enhanced PD-L1 expression induced by SBRT could make patients more susceptible to subsequent PD-1/PD-L1 inhibitors, which further attains a high response rate and extends overall survival. Of particular interest, increased tumor burden is associated with a decreased efficacy of PD-1 inhibitors [56]. SBRT could reduce disease burden through direct killing effects and indirect immune response, which further improves the outcomes of PD-1/PD-L1 inhibitors.

PD-1/PD-L1 inhibitors can attenuate radioresistance and boost abscopal effects. A study conducted by Deng et al. showed that PD-1/PD-L1 inhibitors played an important role in reducing the local accumulation of MDSCs induced by radiation through the cytotoxic actions of tumor necrosis factor α (TNFα) [41]. The restoration of CD8+ T cells after PD-1/PD-L1 inhibitor treatment induced the generation of TNFα, which further led to the elimination of MDSCs. In addition, PD-1/PD-L1 inhibitors can boost the abscopal effect by further enhancing the immune response and breaking the emerging limits of SBRT [57]. Of note, PD-1/PD-L1 inhibitors could improve the deficiency of SBRT in not being able to produce durable antitumor effects. It has been shown that treatment with hypofractionated radiotherapy alone was unable to generate durable antitumor immunity, while it combined with PD-1/PD-L1 inhibitors that can induce protective immunologic memory in long-term survivors with increased memory CD8+ T cells [41].

## Current advances in SBRT and PD-1/PD-L1 inhibitors for NSCLC treatment

### Advances in SBRT for NSCLC treatment

Recently, advances in SBRT for NSCLC treatment have been promising with acceptable safety profiles. SBRT can accurately deliver high doses with relatively small radiation fractions to target tumors, resulting in minimal damage to surrounding healthy tissues. Compared to conventional radiotherapy, the CHISEL trial demonstrated that NSCLC patients with SBRT treatment showed significantly improved local control rates and overall survival (OS) rates (2-year local failure rates, 10% vs. 40%) [19]. In addition, a propensity-matched cohort of 497 patients showed that SBRT is associated with improved local failure rates and 3-year OS rates compared with conventional radiotherapy (13.6% vs. 34.1% and 53.1% vs. 38.9%, respectively) [20]. Currently, it is well accepted that SBRT has become the best alternative to surgery in early-stage, medically inoperable NSCLC patient s[15]. Moreover, emerging evidence indicates that SBRT and definitive surgery may not significantly differ in survival outcomes for patients with medically operable, early-stage NSCLC [16]. Further, larger direct comparisons of the two therapies are warranted to facilitate the selection of the optimal treatment regimen for patients. Except for early-stage NSCLC, SBRT also exhibited great potential in the treatment of advanced oligometastatic NSCLC [17]. Two published phase 2 trials both demonstrated that consolidative SBRT prior to maintenance chemotherapy could almost triple the progression-free survival (PFS) rate in patients with oligometastatic NSCLC when compared with maintenance chemotherapy alone, with no significant differences in adverse effects (11.9 months vs. 3.9 months, 9.7 months vs. 3.5 months) [58, 59]. Of particular concern, though SBRT targeting macroscopic oligometastases could achieve appreciable local control, the development of new metastatic lesions after SBRT monotherapy is still very likely, which is mainly attributed to unirradiated microscopic oligometastases [60]. This indicates that SBRT combined with systemic antitumor treatment may achieve superior tumor control and further prolong survival. Of note, based on the immunomodulatory effect and abscopal response of SBRT, the combination of SBRT with immunotherapy appears to be the most promising treatment protocol for NSCLC.

### Advances in SBRT combined with PD-1/PD-L1 inhibitors for NSCLC treatment

In recent years, preclinical and clinical trials have demonstrated that radiotherapy combined with PD-1/PD-L1 inhibitors can improve immunosuppression and restore CTL responses, thus significantly suppressing tumor growth and prolonging patient survival (Table 1).

**Table 1:** Landmark trials of radiotherapy combined with PD-1/PD-L1 inhibitors for the treatment of NSCLC

NCT number	Patients	Tumor stage	PD-1/PD-L1 inhibitor	Radiotherapy planning	Treatment schedule	Outcomes				Reference
						ORR	PFS (months)	OS (months)	AE (3-5)	
NCT01295827	97	Stage IV advanced	Pembrolizumab 10 mg/kg q2w or 10 mg/kg q3w or 2 mg/kg q2w	Previously received any radiotherapy	Pembrolizumab with a history of radiotherapy vs pembrolizumab alone	NR	mPFS 4.4 vs. 2.1; *p* = 0.019	mOS 10.7 vs. 5.3; *p* = 0.026	Treatment-related pulmonary toxicity 13% vs. 1%	58
NCT02343952	92	Stage III	Pembrolizumab 200 mg q3w for up to 1 year	59–66.6 Gy radiotherapy	Concurrent chemoradiation with consolidation pembrolizumab	NR	mPFS 15.4 m 12, 18, and 24-month PFS were 59.9%, 49.5%, and 45.4% respectively	Estimates of 12 and 24-months OS were 80.5% and 68.7% respectively	G ≥ 2 pneumonitis 17.2%; G3-4 pneumonitis 5.4%, no other G3/4 toxicities exceeded 5%	59
NCT02125461	709	Stage III	Durvalumab 10 mg/kg q2w for up to 12 months	Previously definitive chemoradiotherapy	Durvalumab + previous chemoradiotherapy vs placebo + previous chemoradiotherapy	28.4% vs. 16.0%; *p* < 0.001	mPFS 16.8 vs. 5.6; *p* < 0.001	mOS 23.2 vs. 14.6; *p* < 0.001	29.9% vs. 26.1%	60
NCT02621398	21	Stage III	Pembrolizumab 200 mg q3w or 100 mg q3w	Concurrent chemoradiotherapy (60 Gy in 30 fractions)	Pembrolizumab + concurrent chemoradiotherapy	NR	mPFS with at least 1 dose of pembrolizumab 18.7 m; mPFS with at least 2 doses of pembrolizumab 21 m	mOS 29.4 m	NR	62
NCT02608385	79	Advanced Solid Tumors	Pembrolizumab 200 mg q3w	SBRT 30 to 50 Gy in 3 to 5 fractions	Pembrolizumab + multisite SBRT	13.2%	mPFS 3.1 m	mOS 9.6 m	DLT rate 9.7%	29
NCT02492568	92	Advanced	Pembrolizumab 200 mg/kg q3w	24 Gy in 3 fractions	Pembrolizumab alone vs. pembrolizumab + SBRT	18% vs. 36%; *p* = 0.07	mPFS 1.9 vs 6.6; *p* = 0.19	mOS 7.6 vs. 15.9*; p* = 0.16	NR	63

*ORR* overall response rate, *mPFS* median progression-free survival, *mOS* median overall survival, *AE* adverse effect, *DLT* dose-limiting toxicity

Preclinical studies have shown that hypofractionated radiotherapy in combination with PD-1/PD-L1 inhibitors could improve the long-term survival rate and protect against tumor recurrence in mouse models of melanoma, colorectal cancer, breast cancer, and NSCLC [41, 42, 61]. For clinical trials of conventional radiotherapy combined with PD-1/PD-L1 inhibitors, in a secondary analysis of the phase 1 KEYNOTE-001 study, the PD-1 inhibitor pembrolizumab was demonstrated to confer a longer PFS and OS in patients who previously received any radiotherapy than in those without prior radiotherapy (mPFS, 4.4 vs 2.0 months; OS, 10.7 vs 5.3 months; *p* = 0.034) [62]. The further phase 2 Hoosier Cancer Research Study administering consolidative pembrolizumab after chemoradiotherapy in patients with unresectable stage III NSCLC updated their results in the 2018 ASCO [63]. The mPFS was 15.4 months, with 12-, 18-, and 24-month PFS rates of 59.9%, 49.5%, and 45.4%, respectively. Similarly, the phase 3 PACIFIC study compared the PD-L1 inhibitor durvalumab as consolidation therapy with placebo in patients with advanced NSCLC who did not have disease progression after two or more cycles of chemoradiotherapy [64]. Durvalumab treatment resulted in a longer mPFS (16.8 months vs. 5.6 months) and a higher overall response rate (ORR) (28.4% vs. 16.0%; *p* < 0.001) than placebo. The ETOP NICOLAS trial conducted by Peters et al. demonstrated the treatment feasibility of concurrent nivolumab combined with chemoradiotherapy in unresectable stage III NSCLC with manageable toxicity [65]. Recently, a nonrandomized controlled phase 1 trial was performed in patients with locally advanced unresectable stage III NSCLC who were treated with pembrolizumab and concurrent chemoradiotherapy. The concurrent incorporation resulted in a promising PFS of 69.7% at 12 months with generally well-tolerated toxic effects [66]. Partial response (PR) accounted for 74%, with complete response (CR) achieved in 16% and stable disease (SD) in 5%. The results of further ongoing phase 2 trial are worth looking forward to (ClinicalTrials.gov. NCT03631784).

In view of the increasing number of studies confirming the efficacy of SBRT over conventional radiotherapy, based on the above research findings, clinical trial studies began to explore whether SBRT combined with PD-1/PD-L1 inhibitors can achieve substantially improved clinical benefits. A phase I study estimated multisite SBRT followed by pembrolizumab for metastatic solid tumors, including NSCLC [39]. Patients enrolled in this trial received standard SBRT to two to four metastases with dosing ranging from 30 to 50 Gy in three to five fractions. Pembrolizumab was administered within 7 days after the final SBRT fraction. The combination treatment achieved high control rates in irradiated tumors and responses in nonirradiated metastases. The RECIST-based overall ORR was 13.2%. Moreover, the mOS and mPFS were 9.6 months (95% CI, 6.5 months to undetermined) and 3.1 months (95% CI, 2.9 to 3.4 months), respectively [39]. Likewise, a multicenter, randomized phase 2 study was conducted to evaluate whether SBRT on a single tumor site prior to pembrolizumab treatment potentiated antitumor immune response in patients with metastatic NSCLC [67]. In this study, the enrolled patients were randomized to the control arm (*n* = 40) or the experimental arm (*n* = 36) with treatment scheduling of pembrolizumab either alone (control arm) or after SBRT (3 doses of 8 Gy) (experimental arm). The results showed that patients treated with SBRT combined with pembrolizumab had better mPFS and mOS rates than those treated with pembrolizumab alone (6.6 months vs. 1.9 months, *p* = 0.19 and 15.9 months vs. 7.6 months, *p* = 0.16, respectively). In particular, patients with PD-L1-negative NSCLC were observed to attain the greatest benefit from combination treatment, with statistically significant differences in mPFS and mOS [67]. Besides, the 2019 ASTRO published a phase II prospective trial result in patients with metastatic NSCLC who received SBRT after progression on pembrolizumab treatment [68]. Of the 56 patients enrolled in this trial, 21 patients suffered from disease progression after pembrolizumab monotherapy and received SBRT treatment. The final result reported that the addition of SBRT after progression on the PD-1 inhibitor led to increased PFS with a systemic response rate of 9.52% and a disease control rate of 57.14%. Notably, the substantially improved PFS was associated with an increased TIL score, the presence of an immune-related adverse event, and relative T cell activation status. Moreover, a phase I study conducted by Kelly et al. explored the efficacy and safety of atezolizumab plus SBRT for patients with medically inoperable early-stage NSCLC [69]. The preliminary results updated in the 2020 ASCO indicated that the combination treatment was a feasible choice with no significant additional toxicities. In addition, favorable efficacy is worth expecting in further a randomized phase III trial SWOG/NRG S1914. Except for single immune checkpoint inhibitors combined with SBRT, dual checkpoint inhibitors with SBRT showed impressive tumor control and survival benefits as well. A phase I trial has been performed to evaluate concurrent or sequential ipilimumab, nivolumab, and SBRT in patients with stage IV NSCLC. The latest updated data showed that the mPFS was 5.9 months in the sequential arm and 6.2 months in the concurrent arm with RECIST best response 11% CR, 57% PR, and 6% SD [70].

Although relatively few studies have been completed, the impressive efficacy has garnered substantial enthusiasm to develop and conduct more clinical trials. The ongoing clinical trials investigating SBRT combined with PD-1/PD-L1 inhibitors in patients with various stages of NSCLC are presented in Table 2. Among the 24 trials, the most common study phases were phase 1 and 2, with only 3 phase 3 trials. Except for stage IV NSCLC, an increasing number of trials are focusing on early-stage NSCLC to try to expand the indication. For the SBRT regimen, there is still a lack of consensus, with ranges from 30 Gy to 60 Gy in 3 to 5 fractions. Pembrolizumab and durvalumab are the two most commonly used PD-1/PD-L1 inhibitors in ongoing trials, which also include nivolumab, toripalimab, sintilimab, atezolizumab, and avelumab. The primary outcomes mainly focus on the efficacy and safety of the combination treatment, involving ORR, PFS, OS, relapse-free survival (RFS), event-free survival (EFS), time to progression (TTP), and toxicity rate, with only one exploring the change in the number of infiltrating CD3+ T cells in lesion biopsy. In summary, ongoing trials will further help us obtain a comprehensive understanding of the combination treatment, thus guiding clinical practice to achieve superior survival benefits.

**Table 2:** Ongoing trials of SBRT in combination with PD-1/PD-L1 inhibitors in NSCLC treatmen**t**

NCT number	Phase	NSCLC stage	SBRT regimen	PD-1/PD-L1 inhibitors	Trial design (Arms)	Primary outcome	Notes
NCT03050554	Phase 1, Phase 2	Early stage	12 Gy × 4 fractions or 10 Gy × 5 fractions over 10–12 days every other day	Avelumab 10 mg/kg q2w for 6 cycles	SBRT + avelumab	Safety and tolerability of the combination treatment; RFS	To investigate the efficacy of SBRT combined with avelumab in the treatment for early stage NSCLC
NCT03924869	Phase 3	medically inoperable stage I or IIA	45–54 Gy/3–5 fractions over approximately 2 weeks every 3 days	Pembrolizumab 200 mg q3w for up to 17 cycles	Experimental: SBRT + pembrolizumab Placebo comparator: SBRT + placebo	EFS (up to approximately 6 years); OS (up to approximately 6 years)	To explore the efficacy and safety of SBRT plus pembrolizumab in the treatment of medically inoperable Stage I or IIA NSCLC.
NCT03383302	Phase 1, Phase 2	Stage I and II	18 Gy × 3 fractions or 11 Gy × 5 fractions	Nivolumab 240 mg q2w for up to 1 year	Nivolumab + SBRT	Assessment of lung toxicity (pneumonitis)[6 months from final dose of SBRT administered for each patient ]	To assess the lung toxicities from treatment with nivolumab after SBRT for early stage NSCLC
NCT03574220	Phase 1	Medically inoperable early stage	50 Gy in 5 fractions over 5–14 days, or 60 Gy in 3 fractions over 8–15 days.	Pembrolizumab 200 mg q3w for up to 6 months	Pembrolizumab + SBRT	Percent of patients tolerant to study drug (up to 12 months)	To explore the efficacy of SBRT combined with pembrolizumab in the treatment of medically inoperable early stage NSCLC
NCT02599454	Phase 1	Stage I	50Gy in 4 fractions for peripherally located tumors and 50 Gy in 5 fractions for centrally located tumors	Atezolizumab Courses repeat every 3 weeks	Atezolizumab + SBRT	Maximum tolerated dose (9 weeks)	To investigate the toxicities and best dose of atezolizumab that can be given together with SBRT in treating patients with stage I NSCLC that cannot be removed by surgery
NCT03217071	Phase 2	stage I–IIIA	12 Gy in 1 fraction	Pembrolizumab 200 mg q3w for 2 cycles	Pembrolizumab vs. pembrolizumab + SBRT	Change in number of infiltrating CD3+ T cells/μm2	To determine whether neoadjuvant pembrolizumab +/− SRT is sufficient to produce a two-fold change in the CD3+ T cell population, comparing pre-treatment biopsy tissue to post-treatment resection specimens
NCT03436056	Phase 1	Stage IV	30 Gy in 3 fractions, 54 Gy in 3 fractions, the maximum tolerated dose determined before	Pembrolizumab 200 mg q3w	Dose escalation cohort 1, SBRT 30 Gy 3 fractions + pembrolizumab Dose escalation cohort 2, SBRT 54 Gy 3 fractions + pembrolizumab Expansion cohort, maximum tolerated dose determined before +p embrolizumab	Toxicity rate, (12 weeks from the last dose of lung SBRT) establish the recommended dose of SBRT (12 weeks from the last dose of lung SBRT)	To explore the safety of SBRT combined with pembrolizumab and establish the recommended dose for phase 2 trials of lung SBRT that can be safely combined with pembrolizumab.
NCT03867175	Phase 3	Stage IV	3–10 treatments of SBRT	Pembrolizumab 200 mg q3-4w for up to 1 year	Experimental arm, SBRT + pembrolizumab Control arm, Pembrolizumab alone	PFS (up to 5 years)	To explore how well SBRT combined with immunotherapy works compared with immunotherapy alone after first-line systemic therapy in patients with stage IV NSCLC
NCT02904954	Phase 2	Stage I, II, and IIIA	SBRT delivered in 3 daily fractions	Durvalumab	Experimental arm, Durvalumab + SBRT Control arm, Durvalumab alone	Disease-free survival (up to 26 months)	To find out the effectiveness of durvalumab with or without SBRT as treatment for stage I, II, and IIIA NSCLC prior to surgery and 1 year following surgery
NCT03589547	Phase 2	Stage III	20 Gy in 2 fractions	Durvalumab 10 mg/kg q2w for up to 1 year	Durvalumab + SBRT	Number of patients experiencing grade 2 or higher toxicities during combination therapy (the first 3 months of durvalumab) Average PFS (for about 5 years)	To investigate the safety and efficacy of the combination of durvalumab and SBRT.
NCT03148327	Phase 1, Phase 2	non-metastatic, early stage	54 Gy in 3 fractions or 50 Gy in 4 fractions or 65 Gy in 10 fractions	Durvalumab 1500 mg q4w for up to 4 cycles	SBRT + durvalumab SBRT alone	treatment-related adverse events as assessed by CTCAE v4.0(4 months), mPFS (2 years)	To explore the safety and efficacy of the combination of durvalumab and SBRT vs. SBRT alone
NCT03110978	Phase 2	Stage I, selected stage IIa or isolated	SBRT	Nivolumab For up to 12 weeks	SBRT alone SBRT + nivolumab	Event-free survival (EFS) [2 years]	To investigate the efficacy of SBRT combined with nivolumab in patients with stage I–IIA NSCLC
NCT03446547	Phase 2	Stage I	SBRT 3–4 fractions	Durvalumab 1500 mg q4w for up to 1 year	Arm A, SBRT Arm B, SBRT + durvalumab	Time to progression (TTP)	To explore the efficacy of SBRT combined with durvalumab in patients with stage I NSCLC
NCT03833154	Phase 3	Early stage	SBRT	Durvalumab 1500 mg q4w for up to 2 year	Experimental arm, Durvalumab + SBRT Control arm, Placebo + SBRT	PFS (up to 5 years)	To assess the efficacy and safety of durvalumab versus placebo following SBRT in patients with unresected Stage I/II lymph node-negative NSCLC.
NCT02407171	Phase 1, Phase 2	Stage IV	30 Gy in 5 fractions, 30 Gy in 3 fractions, 10 Gy in 1 fraction	Pembrolizumab 200 mg q2w	SBRT + pembrolizumab	ORR (up to 12 months) Dose-limiting toxicity (up to 12 months)	To explore the efficacy and safety of SBRT combined with pembrolizumab in metastatic NSCLC.
NCT02444741	Phase 1, Phase 2	Stage IV	50 Gy in 4 fractions or 45 Gy in 15 fractions	Pembrolizumab 200 mg q2w	SBRT + pembrolizumab	ORR; incidence of toxicity; maximum tolerated dose of pembrolizumab and SBRT	To explore the efficacy and safety of SBRT combined with pembrolizumab in stage IV NSCLC. The research also aims to compare different types of radiotherapy.
NCT02608385	Phase 1	Stage IV	3 or 5 doses of SBRT to the chosen metastases	Pembrolizumab 200 mg q3w	SBRT + pembrolizumab	Recommended SBRT dose in combination with Pembrolizumab.	To evaluate the safety of SBRT combined with pembrolizumab and determine the safe doses of radiation when used together with pembrolizumab.
NCT02658097	Phase 2	Stage IV	8 Gy in 1 fraction	Pembrolizumab 200 mg q3w	SBRT + Pembrolizumab	ORR	To explore the efficacy of SBRT combined with pembrolizumab with some focus on the tumor responses outside the radiation field.
NCT02492568	Phase 2	Stage IV	24 Gy in 3 fractions	Pembrolizumab 200 mg q3w for up to 2 years	SBRT + pembrolizumab vs. pembrolizumab alone	ORR	To evaluate the increase in ORR in the pembrolizumab alone arm compared to the pembrolizumab after SBRT arm at 12 weeks
NCT03812549	Phase 1	Stage IV	30 Gy in 3 fractions	Sintilimab 200 mg q3w for up to 2 years	SBRT + low dose radiotherapy (LDRT) dose from 2 to 10 Gy + sintilimab vs. SBRT + LDRT dose at MTD determined + sintilimab	Number of participants with adverse events and dose limiting toxicities	To investigate the safety and tolerability of sintilimab in combination with concurrent SBRT and low dose radiotherapy in patients with stage IV NSCLC
NCT03275597	Phase 1	Stage IV	30 and 50 Gy in five fractions over 2 weeks	Durvalumab 1500 mg q4w Tremelimumab 75 mg q4w	SBR + durvalumab + tremelimumab	Safety and tolerability	To evaluate safety and tolerability of dual checkpoint inhibition of durvalumab and tremelimumab with SBRT in the treatment of oligometastatic NSCLC and to examine the sequential delivery of SBRT to all disease sites followed by combination of durvalumab and tremelimumab.
NCT04238169	Phase 2	Stage IV	30–50 Gy in 5 fractions	Toripalimab 240 mg q3w for 9 cycles	SBRT + toripalimab vs. SBRT + Bevacizumab + toripalimab	ORR	To investigate the effect of SBRT and immunotherapy combined with bevacizumab or not in stage IV NSCLC with previously failed after chemotherapy.
NCT04255836	Phase 2	oligo-metastatic	50–60 Gy/≤ 10 fractions	Durvalumab 1500 mg q3w for 4 cycles and 1500 mg q4w for 2 years	Durvalumab + chemotherapy + SBRT	PFS	To assess the efficacy and safety of durvalumab combined with chemotherapy and SBRT in patients with oligo-metastatic NSCLC
NCT03955198	Phase 2	Advanced NSCLC with 1 to 4 brain metastases	SBRT	Durvalumab	SBRT vs. SBRT + durvalumab	Time to intra-cranial progression	To evaluate whether the combination of SBRT with durvalumab in patients with brain metastases from NSCLC improves brain tumor control compared to SBRT alone.

*NSCLC* non-small cell lung cancer, *SBRT* stereotactic body radiotherapy, *SABR* stereotactic ablative radiotherapy, *RFS* relapse free survival, *EFS* event-free survival, *OS* overall survival, *PFS* progression-free survival, *TTP* time to progression, *ORR* overall response rate

## Future challenges and directions for SBRT combined with PD-1/PD-L1 inhibitors

### The optimal radiation dose and fractionation

Despite two decades of increasingly widespread use, there is still no clear consensus on the recommended standard dose and fractionation of SBRT in clinical practice. Current preclinical and clinical trials have studied several regimens with no conclusion as to which is optimal.

To date, available SBRT regimens for early-stage NSCLC involve 30–34 Gy × 1 fraction, 15–20 Gy × 3 fractions, 12 Gy × 4 fractions, and 10–12 Gy × 5 fractions, with the most common overall being the latter two [71]. A randomized phase II trial showed that 30 Gy in one fraction was equivalent to 60 Gy in three fractions with regard to toxicity, local control, PFS, and OS in the treatment of peripheral stage I-II NSCLC [72]. Similarly, the phase 2 RTOG 0915 study also found that a single fraction of 34 Gy and 48 Gy in 4 fractions achieved similar 5-year primary tumor control rates, with the single fraction regimen leading to slightly fewer grade 3 or higher adverse events [73, 74]. Stephans et al. conducted a retrospective study of NSCLC treated with definitive intent SBRT, demonstrating that the SBRT regimen of 54–60 Gy in 3 fractions attained a statistically significant lower local failure (4.3% at 2 years) than 30–34 Gy in 1 fraction (21%), 48–50 Gy in 4–5 fractions (15.5%), and 50–60 Gy in 8–10 fractions (13.3%) [75]. Notably, the high-dose 3-fraction regimen was associated with slightly higher but tolerable pulmonary and chest wall toxicities than the other regimens. In addition, currently limited data have shown that when combined with PD-1/PD-L1 inhibitors, SBRT doses vary from 30 to 50 Gy in 3 to 5 fractions with acceptable toxicity [39]. The PEMBRO-RT phase 2 randomized clinical trial demonstrated that an SBRT dose of 8 Gy × 3 fractions could significantly enhance the antitumor immunity of pembrolizumab with improved ORR, PFS, and OS [67].

The selection of the optimal radiation dose and fractionation should, on the one hand, ensure adequate priming of antitumor immunity and, on the other hand, minimize the occurrence of adverse effects as much as possible. We think that the discrepancy in the optimal radiation dose and fractionation might partly be attributed to different tumor pathological types, tumor sizes, tumor locations, metastatic states, intrinsic radiosensitivity, and host characteristics, which makes it difficult to directly compare the different studies and determine the standard regimen. Based on the currently limited data, we speculate that varying optimal doses and fractionation might exist for patients with multimodal tumor characteristics and various immune states. In the era of precision medicine, further analysis is warranted to determine whether subgroups of patients with clear benefits from varying optimal doses and fractionation.

### The optimal schedule for combining SBRT and PD-1/PD-L1 inhibitors

Currently, the optimal schedule for combining SBRT and PD-1/PD-L1 inhibitors remains to be fully elucidated and might have a significant effect on the generation of a potent and durable antitumor immune response. Multiple treatment modalities have been explored over the past few decades focusing on the optimal schedule, including the sequencing and timing (concurrent vs. sequential) of the combination.

For the optimal sequencing of the combination, there is no clear consensus on whether SBRT should be performed before or after the initiation of PD-1/PD-L1 inhibitors. Generally, based on the immunomodulatory effect of radiation, evidence has demonstrated that SBRT has great potential to sensitize subsequent immunotherapy [13]. Immune activation and the possible conversion of “cold” to “hot” tumors after SBRT lead to a supportive tumor microenvironment for subsequent PD-1/PD-L1 inhibitors [34, 76]. In turn, PD-1/PD-L1 inhibitors could reinvigorate exhausted or resting CTLs, which serves as the basis for SBRT-induced antitumor immunity [51]. A retrospective analysis from two prospective study of nivolumab combined with stereotactic radiosurgery (SRS) in treatment of advanced metastatic melanoma showed that there was no significant difference in OS and local control among the subgroups of SRS either before, during, or after administration of nivolumab [77]. To date, there has been a lack of relevant trials comparing the two schedules head to head. The current treatment combination tends to favor SBRT prior to PD-1/PD-L1 inhibitors. Whether sequencing affects the efficacy and safety of the combination remains to be further investigated.

In an effort to minimize adverse effects, when SBRT is combined with PD-1/PD-L1 inhibitors for tumor treatment, there is a great predisposition to select sequential instead of concurrent combination schedules in clinical research and practice. The originally published KEYNOTE-001 and PACIFIC trials both demonstrated the feasibility and efficacy of sequential radiotherapy combined with PD-1/PD-L1 inhibitors [62, 64]. Nonetheless, considering that previous studies have confirmed that concurrent chemoradiotherapy and concurrent chemotherapy with PD-1/PD-L1 inhibitors achieve superior survival benefits compared with sequential schedules, attention is increasingly being focused on exploring whether the optimal schedule applies to SBRT combined with PD-1/PD-L1 inhibitors [78, 79]. Promisingly, the currently available data of the concurrent incorporation of SBRT and PD-1/PD-L1 inhibitors demonstrated substantially improved PFS and OS with manageable toxicity [39, 67]. In addition, Hettich et al. conducted a detailed tumor-infiltrating lymphocyte (TIL) kinetic evaluation of the tumor microenvironment after hypofractionated radiotherapy of 24 Gy in 2 fractions, observing that there was a transient increase in tumor-specific CD8+ TILs at approximately days 5–8 after radiation, while unexpectedly, suppressive Treg cells dominated around days 10–16 [80]. This finding proposes another challenge regarding the ideal time interval between SBRT and PD-1/PD-L1 inhibitors. Mechanistically, to achieve the best efficacy, the ideal time interval should integrate SBRT-induced immune sensitization with PD-1/PD-L1 inhibitor-induced immune activation.

Overall, the optimal schedule should coincide with enhanced antitumor immunity. Based on the limited available data, we speculate that the sequencing of the combination treatment makes almost no difference in efficacy irrespective of SBRT before or after PD-1/PD-L1 inhibitors. Though with significantly improved survival outcomes, whether it is optimal to concurrently combine SBRT with PD-1/PD-L1 inhibitors possibly at the cost of increased toxicities remains to be further investigated. Prospective head-to-head clinical trials are warranted to provide more solid evidence and further guide clinical research and practice.

### Selection of a suitable irradiated volume and target

Evidence has demonstrated that SBRT could accurately deliver high doses to target lesions with minimal damage to surrounding healthy tissues. Nonetheless, there is still a lack of consensus on the suitable irradiated lesion volume and target to achieve the best efficacy. Moreover, with the increasingly significant benefits of SBRT in oligometastatic NSCLC, there is a growing concern about whether multisite therapy is superior to single-single therapy.

Weighing the efficacy and safety of SBRT treatment, the most suitable irradiated volume remains unclear. Studies have shown that the overall tumor burden may have a substantial correlation with systemic immune responses [56]. Notably, it has been found that increased tumor burdens might give rise to decreased efficacy of PD-1 inhibitors. SBRT at high doses can play a significant role in tumor debulking, which could further pave the way for the administration of PD-1/PD-L1 inhibitors. Of particular concern, generally, a larger irradiated volume could result in a dramatically decreased tumor burden, which allows for further treatment. In addition, the selection of a larger irradiated volume can cover the malignant tissue more comprehensively, thus reducing the recurrence of the primary lesions. However, considering safety, the relatively larger irradiated volume might be limited by critical nearby normal structures, including the bronchial tree, heart, brachial plexus, and esophagus. In addition, the adjacent, potentially affected draining lymphatics should also be considered in the selection of the suitable irradiated volume. Based on previous clinical practice and research experience, it is generally accepted that the anatomic draining lymphatics of primary tumors need to be irradiated with a larger volume to avoid treatment failure involving local failure and regional lymph node recurrence. Nevertheless, a growing body of evidence indicates that the tumor-associated draining lymph node is responsible for the generation and priming of CTLs and further trafficking into the tumor microenvironment [81–84]. The newly exposed tumor neoantigens are delivered to the draining lymph nodes, where they trigger cross-presentation by DCs, thus leading to a potent antitumor immune response [25, 83]. Therefore, instead of sterilizing draining lymphatics with larger irradiated volumes, a relatively smaller irradiated volume might contribute to supportive antitumor immunity with improved survival outcomes, especially when combined with PD-1/PD-L1 inhibitor treatment.

In order to minimize the potential toxicity, for the combination treatment in metastatic NSCLC, priority was initially given to single site irradiation. However, further exploration for potent systemic antitumor immunity suggests that single site irradiation of metastatic NSCLC might not be associated with the sufficient generation, priming, and infiltration of TILs to all lesions. Multisite irradiation is emerging as a paradigm shift to optimize the efficacy of SBRT combined with PD-1/PD-L1 inhibitors. A phase I trial and the phase II multicenter SABR-COMET trial both demonstrated that multisite SBRT followed by PD-1/PD-L1 inhibitors resulted in high local and distant lesion control with well-tolerated toxicities [39, 85]. Increasing evidence has confirmed the feasibility and necessity of multisite irradiation. For one thing, compared with a single site to pursue relatively rare “abscopal” effects, multisite SBRT is likely to stimulate a more robust systemic immune response [86]. Research has revealed that tumor-specific and site-specific immunogenic heterogeneity exists, which indicates that each lesion might require its own specific stimulation; thus, targeting a single site might fail to broadly activate antitumor immunity [87, 88]. Multisite SBRT may facilitate increased antigen release and presentation, which ultimately induces enhanced priming and trafficking of TILs to the corresponding TME. For another, multisite SBRT could achieve a pronounced reduction in overall tumor burden, which contributes to optimizing responses to PD-1/PD-L1 inhibitors [56]. Therefore, in the context of controlled, tolerable toxicity, multisite SBRT has the potential to achieve superior survival benefits, especially when combined with PD-1/PD-L1 inhibitors. Of particular concern, current SBRT delivery is restricted to approximately three isocenters per patient per day, which poses great challenges in treating patients with 4 or more sites of metastatic NSCLC [86]. Further, development of improved facilities that are capable of autonomously contouring, planning, and delivering irradiation all in only one day is urgently needed to achieve more isocenters that can be conducted in one session.

Instead of one-cycle simultaneous multisite SBRT, we consider whether we could achieve better clinical efficacy by completing SBRT of all lesions in multiple cycles with each cycle delivering to one to two lesions. It has been demonstrated that SBRT could stimulate potent antitumor immunity and further provide a supportive tumor microenvironment for PD-1/PD-L1 inhibitors. Nonetheless, the radiation-induced immune activation effect is not sustained. Challenges are posed regarding whether multisite SBRT should be performed in several cycles (“pulsing” regimen) to further consolidate and potentiate the antitumor immune response, thus achieving potent and durable antitumor immunity as well as substantially improved survival outcomes. Besides, current available studies on SBRT combined with PD-1/PD-L1 inhibitors are mostly limited to oligometastatic NSCLC (< 5 sites) while for NSCLC with limited metastases (> 5 sites), whether multisite SBRT or even all-site SBRT combined with PD-1/PD-L1 inhibitors could attain superior survival benefits remains to be explored clinically. For the combination treatment, the selection of a suitable irradiated volume and target of SBRT should not only pursue superior survival benefits but also place great emphasis on potential toxicities. The above analysis indicates that a relatively smaller irradiated volume in one site, and multisite irradiation has a promising perspective in future clinical practice. The “pulsing” regimen of multisite SBRT might possibly achieve potentiated and durable immune activation. Further, prospective clinical trials are warranted to provide more solid evidence and more specific standards.

### Potential predictive biomarkers in the combination treatment

The identification of predictive biomarkers to determine the best benefit population is a prerequisite for the widespread application of specific treatments in clinical practice. Nonetheless, current biomarkers for SBRT combined with PD-1/PD-L1 inhibitors remain unclear.

Since the breakthrough of PD-1/PD-L1 inhibitors in cancer treatment, research on their predictive biomarkers has been continuous. To date, a variety of biomarkers have been observed that can potentially predict survival outcomes, but none have definite criteria or efficacy. Intratumoral PD-L1 expression emerged as the first predictive biomarker to select a suitable population for PD-1/PD-L1 inhibitor treatment [89]. Multiple clinical trials (including KEYNOTE-024, IMpower110, etc.) confirmed that NSCLC patients with high PD-L1 expression tended to achieve better survival benefits when treated with PD-1/PD-L1 inhibitors [3, 90]. Independent of PD-L1 expression, tumor mutation burden (TMB), and reflecting genomic instability, can assess the status of tumor neoantigens and further reveal the immune competence of the tumor microenvironment to some extent [91]. It has been demonstrated that high TMB (≥ 10 mutations/mb) was correlated with favorable survival outcomes with substantially improved PFS and OS in PD-1/PD-L1 inhibitor treatmen t[92, 93]. Mismatch repair (MMR) deficiency and microsatellite instability have also shown valuable predictive power, which shed light on a new direction for gene analysis to precisely predict the efficacy of PD-1/PD-L1 inhibitors [94, 95]. Besides, TILs, especially CTLs and cytokines, could also potentially act as promising biomarkers for inhibitor treatment [93, 96]. With regard to SBRT, exploratory analysis showed that radiomics signatures have great potential as imaging predictive biomarkers, such as 18F-FDG PET/CT radiomics and apparent diffusion coefficient for NSCLC patients treated with SBRT [97–99]. Moreover, research has found that systemic inflammation metrics involving the neutrophil-lymphocyte ratio, platelet-lymphocyte ratio, and lymphocyte-monocyte ratio could exert reliable predictive roles in identifying optimal patients for SBRT treatment [100].

Although the predictive biomarkers above have shown encouraging roles in PD-1/PD-L1 inhibitors or SBRT monotherapy, their effects on combination treatment remain unknown. Furthermore, in view of the uncertainty and variability of current biomarkers, further studies of combined therapeutic biomarkers are warranted to carry out new explorations and establish new practices. Instead of the use of a single biomarker, the integrated tumor microenvironment-based signature with multiple parameters, including CTLs, PD-L1 expression, and TMB, might be associated with a higher value of efficacy prediction [101]. This sheds light on the idea that future research of an optimized predictive paradigm for combination treatment should attach importance to the incorporation of several biomarkers. Notably, in addition to economic considerations, the integrated biomarkers should be independent of each other, which is the basis for further exploration. Besides, the unresolved challenges still exist, including undefined cutoff thresholds, various testing assays, lack of representativeness of the biopsy samples, and repeated invasive biopsy. Therefore, progress in prospective trials is warranted not only to determine the current biomarker standard but also to explore novel approaches, such as circulating tumor cells and/or tumor DNA, in the mode of “liquid biopsy”.

It is universally accepted that SBRT combined with PD-1/PD-L1 inhibitors could elicit synergistic effects. Nonetheless, when SBRT or PD-1/PD-L1 inhibitor monotherapy can achieve significant clinical benefits, the selection of combination treatment at the expense of additional toxicities might be unreasonable. The identification of biomarkers to recognize populations suitable for monotherapy or combination treatment is of great significance. Patients with high PD-L1 expression have been observed to be more susceptible to PD-1/PD-L1 inhibitor monotherapy with substantially improved PFS and OS [3]. PD-L1 expression has become a screening criterion for the use of pembrolizumab in NSCLC treatment. Of particular interest, in a multicenter, randomized phase 2 study, it was observed that patients with PD-L1–negative NSCLC have a much higher response rate to combination treatment than those with PD-L1–positive NSCLC [67]. Statistically, significant differences in OS were found only in the PD-L1-negative subgroup instead of the PD-L1-positive subgroup (HR, 0.48; 95% CI, 0.24–0.99; *p* = 0.046; and HR, 1.4; 95% CI, 0.42–4.66; *P* = 0.58, respectively) [67]. An explanation of the phenomenon is that PD-L1-negative expression can be converted to positive during SBRT treatment, which further has a sensitizing effect on PD-1/PD-L1 inhibitors [42, 76]. This finding indicates that negative PD-L1 expression may be an effective biomarker for screening the most suitable patients for treatment with SBRT combined with PD-1/PD-L1 inhibitors. Similarly, to avoid overtreatment, other biomarkers also need to be further studied.

Up to now, due to a lack of sufficient research evidence, there are no validated predictive biomarkers to identify patients who are prone to responding to the combination of SBRT and PD-1/PD-L1 inhibitors. The factors above might have some potential predictive value but are accompanied by some uncertainty. Further, confirmation and other potential biomarkers remain to be fully investigated.

### Safety

Although SBRT combined with PD-1/PD-L1 inhibitors has promising potential to achieve superior clinical efficacy, the regimen might place patients at risk of incurring increased toxicities, which could restrain its widespread application in clinical research and practice. Hence, further research to determine and characterize the safety of the combination treatment remains to be fully conducted.

Early exploration, including the KEYNOTE 001 and PACIFIC trials, both showed that the combination treatment was well tolerated with acceptable toxicities in patients with NSCLC [62, 64]. In detail, the secondary analysis of KEYNOTE-001 demonstrated that there was a statistical difference in treatment-related pulmonary toxicities between patients with prior thoracic radiotherapy and those without (13% vs. 1%, *P* = 0.046), while high-grade pulmonary toxicities showed no significant difference [62]. Likewise, the phase 3 PACIFIC study found that the occurrence rate of grade ≥ 3 immune-related adverse events (irAEs), of which pneumonitis accounted for the majority, did not differ significantly between the durvalumab arm and the placebo arm (29.9% vs. 26.1%), with grade ≥ 3 pneumonitis in 3.4% and 2.6%, respectively [64]. Findings from the two trials provided a basis and support for further studies. Except for some case reports [102–104], several prospective and retrospective studies irrespective of single arm or multiple arms, randomized, or nonrandomized have been conducted. A multicenter safety and toxicity analysis showed that the associated subacute grade ≥ 3 irAEs in patients with SBRT in combination with ICIs or SBRT monotherapy were 26.8% and 2.9%, respectively. The risks of any grade pneumonitis were similar in the two groups (33.9% vs 27.9%, *p* = 0.47), with a significant difference in grade ≥ 3 pneumonitis (10.7% vs 0%, *p* < 0.01) [105]. A phase 1 study with multisite SBRT and pembrolizumab treatment as well as the PEMBRO-RT phase 2 randomized trial showed concordant results with tolerable irAEs [39, 67]. Notably, the combination treatment could also result in fatal irAEs, which should receive more attention. In a meta-analysis, the toxicity-related fatality rates were 0.36% in patients treated with PD-1 inhibitors and 0.38% in those treated with PD-L1 inhibitors [106]. The corresponding fatal irAEs mainly included pneumonitis (35%), hepatitis (22%), and neurotoxicity (15%). The combination regimen might induce overlapping damage, thus leading to the higher occurrence of fatal irAEs than that with monotherapy. With limited data on fatal irAEs, further analysis to explore the potential predictive or affecting factors of fatal toxicities is warranted.

Of particular concern, accumulating research has revealed that there is a potential possibility that the improved survival duration and outcomes might be coupled to the development of irAEs. Several published trials have shown that the occurrence of irAEs might be associated with substantially improved ORR, PFS, and OS in patients with NSCLC who were treated with PD-1/PD-L1 inhibitor monotherapy [107–109]. This predisposition is found in the combination treatment as well. In a retrospective analysis of 201 patients treated with nivolumab combined with prior thoracic radiotherapy, improved mPFS and lower disease progression rates were found in patients with a history of treatment-related pneumonitis compared with those with no such history (3.6 vs. 2.3 months, *p* = 0.023; 29.4% vs. 47.9%, *p* = 0.059) [110]. Likewise, Hwang et al. demonstrated that patients with grade 2 or higher irAEs, especially pneumonitis, had superior survival benefits [107]. We speculate that the development of irAEs might be related to an overactive immune response, which partly indicates that the combination treatment evokes potent antitumor immunity. Hence, there is a high possibility that the occurrence of irAEs is not only closely related to the overlapping toxicity of combination treatment but also has a predictive role in improved PFS and OS. Despite the data mentioned above, there remains controversy over whether irAEs could predict potent antitumor effects and improved survival. Some large retrospective studies have failed to demonstrate a relationship between irAEs and clinical benefits [111, 112]. Moreover, a subset of patients with severe and even fatal irAEs could develop poor prognosis and even die. The discontinuation of PD-1/PD-L1 inhibitors when severe irAEs occur may affect the therapeutic efficacy as well [64]. Overall, though irAEs might have a promising role in predicting improved clinical efficacy, challenges remain to be solved, including more solid evidence, more specific standards and guidelines, the potential mechanism, whether the management of irAEs could affect their predictive role, and other uncertainties.

Increased DNA damage, redundant TIL and inflammatory cytokine release, and potential overlapping toxicities lead to overactive antitumor immunity and fragile damaged tissue, which mainly manifests as the occurrence of irAEs. More effort is needed to explore the challenges related to irAEs. First, irAEs vary in initial attack, kinetics, and presentation in clinical research and practice. Studies are warranted to explore the potential predictive or affecting factors and to identify which patients are prone to certain irAEs for early prevention and focused monitoring. We speculate that a variety of factors may contribute to the development of irAEs, including tumor stage, pathological type, size and location, baseline irradiated organ status, prior treatments, specific SBRT regimen, certain PD-1/PD-L1 inhibitors, and the patient’s overall status. Further, studies are needed to verify these risk factors with some focus on other unexplored potential factors. Second, although most of the irAEs can be treated relatively well, there are still some irAEs that are refractory to current recommendation standards. These irAEs tend to develop because of poor response to treatment, thus resulting in death. Therefore, more research should focus on the management of current treatment refractory irAEs, which could help improve the quality of life and prolong the survival of patients. Third, according to the current treatment standard, PD-1/PD-L1 inhibitors should be discontinued when grade ≥ 3 irAEs occur [113]. Uncertainty remains regarding whether to recommence PD-1/PD-L1 inhibitor treatment after recovery from irAEs. If PD-1/PD-L1 inhibitor treatment cannot be continued, the best alternative therapy needs to be explored. In conclusion, more robust randomized prospective clinical trials with longer and rigorous follow-ups are warranted to obtain a comprehensive understanding of the undue toxicities from the combination treatment.

### Others

Of particular concern, in addition to the above unresolved clinical challenges, there are other unknown practical considerations that remain to be further investigated. First, the current research on the combination treatment mainly focuses on advanced NSCLC. Evidence has shown that patients with early-stage NSCLC still have an unsatisfactory 5-year survival rates ranging from 30 to 49% after definitive resection or SBRT. Therefore, clinical trials are desperately needed to determine whether combination treatment is more effective than SBRT or surgery alone in patients with early stage NSCLC. Second, regarding the PD-1/PD-L1 inhibitors in combination treatment, several clinical considerations remain to be solved, including the optimal duration of PD-1/PD-L1 inhibitor treatment post SBRT to achieve a durable and effective antitumor response, whether the dosing regimen should be adjusted based on monotherapy dosing to minimize the potentially increased toxicities, and whether there is a certain PD-1/PD-L1 inhibitor that can attain the optimal survival outcomes when combined with SBRT. Third, different treatment regimens have different mechanisms of action, which might partly change the physiological and pathological state of the body. Further research is needed to determine whether patients’ previous treatment regimens affect the efficacy and safety of combination treatment. Fourth, clinical trials are warranted to identify the potential beneficial population. In addition to the study population in clinical trials, patients with pre-existing autoimmune diseases, chronic viral infections, organ dysfunction, or other underlying diseases should also be given more attention to explore whether the benefits they can receive outweigh the potential excess toxicities from the combination treatment.

## Conclusion

Advances in PD-1/PD-L1 inhibitors have resulted in a paradigm shift in the management of patients with NSCLC. Nonetheless, immune escape and immune resistance limit their antitumor effect in the majority of patients. The noninvasive, well-tolerated SBRT could substantially modulate the tumor microenvironment to further make tumors more sensitive to PD-1/PD-L1 inhibitors, which depicts a promising landscape of the synergistic combination treatment for NSCLC. To our knowledge, the published data, though limited, indicate that the combination treatment has considerable promise in future NSCLC treatment. Critically, before the extensive application of this combination protocol in clinical practice, more preclinical and clinical trials are urgently needed to provide definite evidence and resolve the challenges discussed above.

## Data Availability

Not applicable.

## Abbreviations

PD-1: Programmed cell death 1
PD-L1: Programmed cell death ligand-1
NSCLC: Non-small-cell lung cancer
SBRT: Stereotactic body radiation therapy
ROS: Reactive oxygen species
ICD: Immunogenic cell death
DCs: Dendritic cells
HMGB1: High-mobility group box 1 protein
TAAs: Tumor associated antigens
dsDNA: Double stranded DNA
cGAS: Cyclic GMP-AMP synthase
STING: Stimulator of interferon genes
IFN: Interferon
TGFβ: Transforming growth factor β
Tregs: Regulatory T cells
TAMs: Tumor-associated macrophages
SDF-1: Stromal cell-derived factor 1
CSF-1: Colony-stimulating factor 1
MDSCs: Myeloid-derived suppressor cells
GMC-SF: Granulocyte-macrophage colony-stimulating factor
NK cells: Natural killer cells
APCs: Antigen presenting cells
TNFα: Tumor necrosis factor α
mOS: Median overall survival
mPFS: Median progression free survival
ORR: Overall response rate
PR: Partial response
CR: Complete response
SD: Stable disease
RFS: Relapse free survival
EFS: Event-free survival
TTP: Time to progression
DAMP: Death-associated molecular patterns
TLR: Toll-like receptors
CTLs: Cytotoxic T lymphocytes
SRS: Stereotactic radiosurgery
TIL: Tumor-infiltrating lymphocyte
TMB: Tumor mutation burden
MMR: Mismatch repair
irAEs: Immune-related adverse events

## References

[1] Torre LA, Siegel RL, Ward EM, Jemal A (2016). Global cancer incidence and mortality rates and trends--an update. Cancer Epidemiol Biomark Prev.

[2] Siegel RL, Miller KD, Jemal A (2019). Cancer statistics, 2019. CA Cancer J Clin.

[3] Reck M, Rodríguez-Abreu D, Robinson AG (2016). Pembrolizumab versus chemotherapy for PD-L1-positive non-small-cell lung cancer. N Engl J Med.

[4] Socinski MA, Jotte RM, Cappuzzo F (2018). Atezolizumab for first-line Treatment of metastatic nonsquamous NSCLC. N Engl J Med.

[5] Besse B, Adjei A, Baas P (2014). 2nd ESMO Consensus conference on lung cancer: non-small-cell lung cancer first-line/second and further lines of treatment in advanced disease. Ann Oncol.

[6] Gandhi L, Rodríguez-Abreu D, Gadgeel S (2018). Pembrolizumab plus chemotherapy in metastatic non-small-cell lung cancer. N Engl J Med.

[7] Langer CJ, Gadgeel SM, Borghaei H (2016). Carboplatin and pemetrexed with or without pembrolizumab for advanced, non-squamous non-small-cell lung cancer: a randomised, phase 2 cohort of the open-label KEYNOTE-021 study. Lancet Oncol.

[8] Herbst RS, Baas P, Kim DW (2016). Pembrolizumab versus docetaxel for previously treated, PD-L1-positive, advanced non-small-cell lung cancer (KEYNOTE-010): a randomised controlled trial. Lancet..

[9] Borghaei H, Paz-Ares L, Horn L (2015). Nivolumab versus docetaxel in advanced nonsquamous non-small-cell lung cancer. N Engl J Med.

[10] Brahmer J, Reckamp KL, Baas P (2015). Nivolumab versus docetaxel in advanced squamous-cell non-small-cell lung cancer. N Engl J Med.

[11] Rittmeyer A, Barlesi F, Waterkamp D (2017). Atezolizumab versus docetaxel in patients with previously treated non-small-cell lung cancer (OAK): a phase 3, open-label, multicentre randomised controlled trial [published correction appears in Lancet. 2017 Apr 8;389(10077):e5]. Lancet..

[12] Qiao M, Jiang T, Ren S, Zhou C (2018). Combination strategies on the basis of immune checkpoint inhibitors in non-small-cell lung cancer: where do we stand?. Clin Lung Cancer.

[13] Sharabi AB, Lim M, DeWeese TL, Drake CG (2015). Radiation and checkpoint blockade immunotherapy: radiosensitisation and potential mechanisms of synergy. Lancet Oncol.

[14] Reynders K, Illidge T, Siva S, Chang JY, De Ruysscher D (2015). The abscopal effect of local radiotherapy: using immunotherapy to make a rare event clinically relevant. Cancer Treat Rev.

[15] Ettinger DS, Wood DE, Aisner DL (2017). Non-small cell lung cancer, Version 5.2017, NCCN Clinical Practice Guidelines in Oncology. J Natl Compr Cancer Netw.

[16] Chang JY, Senan S, Paul MA (2015). Stereotactic ablative radiotherapy versus lobectomy for operable stage I non-small-cell lung cancer: a pooled analysis of two randomised trials [published correction appears in Lancet Oncol. 2015 Sep;16(9):e427]. Lancet Oncol.

[17] Rusthoven KE, Kavanagh BD, Burri SH (2009). Multi-institutional phase I/II trial of stereotactic body radiation therapy for lung metastases. J Clin Oncol.

[18] Timmerman R, Paulus R, Galvin J (2010). Stereotactic body radiation therapy for inoperable early stage lung cancer. JAMA..

[19] Senan S, Rusthoven CG, Slotman BJ, Siva S (2018). Progress in radiotherapy for regional and oligometastatic disease in 2017. J Thorac Oncol.

[20] von Reibnitz D, Shaikh F, Wu AJ (2018). Stereotactic body radiation therapy (SBRT) improves local control and overall survival compared to conventionally fractionated radiation for stage I non-small cell lung cancer (NSCLC). Acta Oncol.

[21] Schaue D, Ratikan JA, Iwamoto KS, McBride WH (2012). Maximizing tumor immunity with fractionated radiation. Int J Radiat Oncol Biol Phys.

[22] Reits EA, Hodge JW, Herberts CA (2006). Radiation modulates the peptide repertoire, enhances MHC class I expression, and induces successful antitumor immunotherapy. J Exp Med.

[23] Chakraborty M, Abrams SI, Camphausen K (2003). Irradiation of tumor cells up-regulates Fas and enhances CTL lytic activity and CTL adoptive immunotherapy. J Immunol.

[24] Formenti SC, Demaria S (2009). Systemic effects of local radiotherapy. Lancet Oncol.

[25] Sharabi AB, Nirschl CJ, Kochel CM (2015). Stereotactic radiation therapy augments antigen-specific PD-1-mediated antitumor immune responses via cross-presentation of tumor antigen. Cancer Immunol Res.

[26] Rapoport BL, Anderson R (2019). Realizing the clinical potential of immunogenic cell death in cancer chemotherapy and radiotherapy. Int J Mol Sci.

[27] Golden EB, Pellicciotta I, Demaria S, Barcellos-Hoff MH, Formenti SC (2012). The convergence of radiation and immunogenic cell death signaling pathways. Front Oncol.

[28] Golden EB, Apetoh L (2015). Radiotherapy and immunogenic cell death. Semin Radiat Oncol.

[29] Weichselbaum RR, Liang H, Deng L, Fu YX (2017). Radiotherapy and immunotherapy: a beneficial liaison?. Nat Rev Clin Oncol.

[30] Jarosz-Biej M, Smolarczyk R, Cichoń T, Kułach N (2019). Tumor microenvironment as a “Game Changer” in cancer radiotherapy. Int J Mol Sci.

[31] Dewan MZ, Galloway AE, Kawashima N (2009). Fractionated but not single-dose radiotherapy induces an immune-mediated abscopal effect when combined with anti-CTLA-4 antibody. Clin Cancer Res.

[32] Deng L, Liang H, Xu M (2014). STING-dependent cytosolic DNA sensing promotes radiation-induced type I interferon-dependent antitumor immunity in immunogenic tumors. Immunity..

[33] Diamond JM, Vanpouille-Box C, Spada S (2018). Exosomes shuttle TREX1-Sensitive IFN-stimulatory dsDNA from irradiated cancer cells to DCs. Cancer Immunol Res.

[34] Burnette BC, Liang H, Lee Y (2011). The efficacy of radiotherapy relies upon induction of type i interferon-dependent innate and adaptive immunity. Cancer Res.

[35] Frey B, Rückert M, Deloch L (2017). Immunomodulation by ionizing radiation-impact for design of radio-immunotherapies and for treatment of inflammatory diseases. Immunol Rev.

[36] Matsumura S, Wang B, Kawashima N (2008). Radiation-induced CXCL16 release by breast cancer cells attracts effector T cells. J Immunol.

[37] Spiotto M, Fu YX, Weichselbaum RR (2016). The intersection of radiotherapy and immunotherapy: mechanisms and clinical implications. Sci Immunol.

[38] Formenti SC, Demaria S (2012). Radiation therapy to convert the tumor into an in situ vaccine. Int J Radiat Oncol Biol Phys.

[39] Luke JJ, Lemons JM, Karrison TG (2018). Safety and clinical activity of pembrolizumab and multisite stereotactic body radiotherapy in patients with advanced solid tumors. J Clin Oncol.

[40] Vanpouille-Box C, Alard A, Aryankalayil MJ (2017). DNA exonuclease Trex1 regulates radiotherapy-induced tumour immunogenicity. Nat Commun.

[41] Deng L, Liang H, Burnette B (2014). Irradiation and anti-PD-L1 treatment synergistically promote antitumor immunity in mice. J Clin Invest.

[42] Dovedi SJ, Adlard AL, Lipowska-Bhalla G (2014). Acquired resistance to fractionated radiotherapy can be overcome by concurrent PD-L1 blockade. Cancer Res.

[43] Wang SC, Yu CF, Hong JH, Tsai CS, Chiang CS (2013). Radiation therapy-induced tumor invasiveness is associated with SDF-1-regulated macrophage mobilization and vasculogenesis. PLoS One.

[44] Xu J, Escamilla J, Mok S (2013). CSF1R signaling blockade stanches tumor-infiltrating myeloid cells and improves the efficacy of radiotherapy in prostate cancer. Cancer Res.

[45] Ruffell B, Chang-Strachan D, Chan V (2014). Macrophage IL-10 blocks CD8+ T cell-dependent responses to chemotherapy by suppressing IL-12 expression in intratumoral dendritic cells. Cancer Cell.

[46] Wirsdörfer F, Cappuccini F, Niazman M (2014). Thorax irradiation triggers a local and systemic accumulation of immunosuppressive CD4+ FoxP3+ regulatory T cells. Radiat Oncol.

[47] Kachikwu EL, Iwamoto KS, Liao YP (2011). Radiation enhances regulatory T cell representation. Int J Radiat Oncol Biol Phys.

[48] Keir ME, Butte MJ, Freeman GJ, Sharpe AH (2008). PD-1 and its ligands in tolerance and immunity. Annu Rev Immunol.

[49] Sharpe AH, Wherry EJ, Ahmed R, Freeman GJ (2007). The function of programmed cell death 1 and its ligands in regulating autoimmunity and infection. Nat Immunol.

[50] Blank C, Gajewski TF, Mackensen A (2005). Interaction of PD-L1 on tumor cells with PD-1 on tumor-specific T cells as a mechanism of immune evasion: implications for tumor immunotherapy. Cancer Immunol Immunother.

[51] Alsaab HO, Sau S, Alzhrani R (2017). PD-1 and PD-L1 checkpoint signaling inhibition for cancer immunotherapy: mechanism, combinations, and clinical outcome. Front Pharmacol.

[52] Garon EB, Rizvi NA, Hui R (2015). Pembrolizumab for the treatment of non-small-cell lung cancer. N Engl J Med.

[53] Gujar S, Pol JG, Kroemer G (2018). Heating it up: Oncolytic viruses make tumors 'hot' and suitable for checkpoint blockade immunotherapies. Oncoimmunology..

[54] Bernstein MB, Krishnan S, Hodge JW, Chang JY (2016). Immunotherapy and stereotactic ablative radiotherapy (ISABR): a curative approach?. Nat Rev Clin Oncol.

[55] Yuan Z, Fromm A, Ahmed KA (2017). Radiotherapy rescue of a nivolumab-refractory immune response in a patient with PD-L1-negative metastatic squamous cell carcinoma of the lung. J Thorac Oncol.

[56] Huang AC, Postow MA, Orlowski RJ (2017). T-cell invigoration to tumour burden ratio associated with anti-PD-1 response. Nature..

[57] Ngwa W, Irabor OC, Schoenfeld JD, Hesser J, Demaria S, Formenti SC (2018). Using immunotherapy to boost the abscopal effect. Nat Rev Cancer.

[58] Gomez DR, Blumenschein GR, Lee JJ (2016). Local consolidative therapy versus maintenance therapy or observation for patients with oligometastatic non-small-cell lung cancer without progression after first-line systemic therapy: a multicentre, randomised, controlled, phase 2 study. Lancet Oncol.

[59] Iyengar P, Wardak Z, Gerber DE (2018). Consolidative radiotherapy for limited metastatic non-small-cell lung cancer: a phase 2 randomized clinical trial. JAMA Oncol.

[60] Tree AC, Khoo VS, Eeles RA (2013). Stereotactic body radiotherapy for oligometastases. Lancet Oncol.

[61] Gong X, Li X, Jiang T (2017). Combined Radiotherapy and Anti-PD-L1 Antibody Synergistically Enhances Antitumor Effect in Non-Small Cell Lung Cancer. J Thorac Oncol.

[62] Shaverdian N, Lisberg AE, Bornazyan K (2017). Previous radiotherapy and the clinical activity and toxicity of pembrolizumab in the treatment of non-small-cell lung cancer: a secondary analysis of the KEYNOTE-001 phase 1 trial [published correction appears in Lancet Oncol. 2017 Jul;18(7):e371]. Lancet Oncol.

[63] Durm GA, Althouse SK, Sadiq AA (2018). Phase II trial of concurrent chemoradiation with consolidation pembrolizumab in patients with unresectable stage III non-small cell lung cancer: Hoosier Cancer Research Network LUN 14-179. J Clin Oncol.

[64] Antonia SJ, Villegas A, Daniel D (2017). Durvalumab after chemoradiotherapy in stage III non-small-cell lung cancer. N Engl J Med.

[65] Peters S, Felip E, Dafni U (2019). Safety evaluation of nivolumab added concurrently to radiotherapy in a standard first line chemo-radiotherapy regimen in stage III non-small cell lung cancer-The ETOP NICOLAS trial. Lung Cancer.

[66] Jabbour SK, Berman AT, Decker RH, et al. Phase 1 Trial of pembrolizumab administered concurrently with chemoradiotherapy for locally advanced non-small cell lung cancer: a nonrandomized controlled trial. JAMA Oncol. 2020;e196731. 10.1001/jamaoncol.2019.6731 PMC7042914 32077891

[67] Theelen WSME, Peulen HMU, Lalezari F (2019). Effect of pembrolizumab after stereotactic body radiotherapy vs pembrolizumab alone on tumor response in patients with advanced non-small cell lung cancer: results of the PEMBRO-RT phase 2 randomized clinical trial. JAMA Oncol.

[68] Campbell AM, Cai WL, Burkhardt D (2019). Final results of a phase II prospective trial evaluating the combination of stereotactic body radiotherapy (SBRT) with concurrent pembrolizumab in patients with metastatic non-small cell lung cancer (NSCLC). Int J Radiat Oncol Biol Phys.

[69] Kelly K, Daly ME, Mirhadi A (2020). Atezolizumab plus stereotactic ablative therapy for medically inoperable patients with early-stage non-small cell lung cancer. J Clin Oncol.

[70] Patel JD, Bestvina CM, Karrison T (2020). Randomized phase I trial to evaluate Concurrent or Sequential Ipilimumab, Nivolumab, and stereotactic body Radiotherapy in patients with stage IV non-small cell lung cancer (COSINR Study). J Clin Oncol.

[71] Yan SX, Qureshi MM, Dyer M, Truong MT, Mak KS (2019). Stereotactic body radiation therapy with higher biologically effective dose is associated with improved survival in stage II non-small cell lung cancer. Lung Cancer.

[72] Singh AK, Gomez-Suescun JA, Stephans KL (2019). One versus three fractions of stereotactic body radiation therapy for peripheral stage I to II non-small cell lung cancer: a randomized, multi-institution, phase 2 trial. Int J Radiat Oncol Biol Phys.

[73] Videtic GM, Hu C, Singh AK (2015). A randomized phase 2 study comparing 2 stereotactic body radiation therapy schedules for medically inoperable patients with stage I peripheral non-small cell lung cancer: NRG Oncology RTOG 0915 (NCCTG N0927). Int J Radiat Oncol Biol Phys.

[74] Videtic GM, Paulus R, Singh AK (2019). Long-term Follow-up on NRG oncology RTOG 0915 (NCCTG N0927): a randomized phase 2 study comparing 2 stereotactic body radiation therapy schedules for medically inoperable patients with stage I peripheral non-small cell lung cancer. Int J Radiat Oncol Biol Phys.

[75] Stephans KL, Woody NM, Reddy CA (2018). Tumor control and toxicity for common stereotactic body radiation therapy dose-fractionation regimens in stage I non-small cell lung cancer. Int J Radiat Oncol Biol Phys.

[76] Twyman-Saint Victor C, Rech AJ, Maity A (2015). Radiation and dual checkpoint blockade activate non-redundant immune mechanisms in cancer. Nature..

[77] Ahmed KA, Stallworth DG, Kim Y (2016). Clinical outcomes of melanoma brain metastases treated with stereotactic radiation and anti-PD-1 therapy. Ann Oncol.

[78] Furuse K, Fukuoka M, Kawahara M (1999). Phase III study of concurrent versus sequential thoracic radiotherapy in combination with mitomycin, vindesine, and cisplatin in unresectable stage III non-small-cell lung cancer. J Clin Oncol.

[79] Aupérin A, Le Péchoux C, Rolland E (2010). Meta-analysis of concomitant versus sequential radiochemotherapy in locally advanced non-small-cell lung cancer. J Clin Oncol.

[80] Hettich M, Lahoti J, Prasad S, Niedermann G (2016). Checkpoint antibodies but not T cell-recruiting diabodies effectively synergize with TIL-inducing γ-irradiation. Cancer Res.

[81] Diamond MS, Kinder M, Matsushita H (2011). Type I interferon is selectively required by dendritic cells for immune rejection of tumors. J Exp Med.

[82] Spranger S, Dai D, Horton B, Gajewski TF. Tumor-residing Batf3 dendritic cells are required for effector T cell trafficking and adoptive T cell therapy. Cancer Cell. 2017;31(5):711-723.e4. 10.1016/j.ccell.2017.04.003 PMC5650691 28486109

[83] Schoenhals JE, Skrepnik T, Selek U, Cortez MA, Li A, Welsh JW (2017). Optimizing radiotherapy with immunotherapeutic approaches. Adv Exp Med Biol.

[84] Marciscano AE, Ghasemzadeh A, Nirschl TR (2018). Elective nodal irradiation attenuates the combinatorial efficacy of stereotactic radiation therapy and immunotherapy. Clin Cancer Res.

[85] Palma DA, Olson R, Harrow S (2019). Stereotactic ablative radiotherapy versus standard of care palliative treatment in patients with oligometastatic cancers (SABR-COMET): a randomised, phase 2, open-label trial. Lancet..

[86] Cushman TR, Gomez D, Kumar R (2018). Combining radiation plus immunotherapy to improve systemic immune response. J Thorac Dis.

[87] Tang C, Welsh JW, de Groot P (2017). Ipilimumab with stereotactic ablative radiation therapy: phase I results and immunologic correlates from peripheral T cells. Clin Cancer Res.

[88] Brooks ED, Chang JY (2019). Time to abandon single-site irradiation for inducing abscopal effects. Nat Rev Clin Oncol.

[89] Davis AA, Patel VG (2019). The role of PD-L1 expression as a predictive biomarker: an analysis of all US Food and Drug Administration (FDA) approvals of immune checkpoint inhibitors. J Immunother Cancer.

[90] Spigel D, de Marinis F, Giaccone G, et al. LBA78IMpower110: Interim overall survival (OS) analysis of a phase III study of atezolizumab (atezo) vs platinum-based chemotherapy (chemo) as first-line (1 L) treatment (tx) in PD-L1–selected NSCLC. Ann Oncol. 2019;30(Supplement_5).

[91] Balar AV, Weber JS (2017). PD-1 and PD-L1 antibodies in cancer: current status and future directions. Cancer Immunol Immunother.

[92] Goodman AM, Kato S, Bazhenova L (2017). Tumor mutational burden as an independent predictor of response to immunotherapy in diverse cancers. Mol Cancer Ther.

[93] Liu SY, Wu YL (2019). Biomarker for personalized immunotherapy. Transl Lung Cancer Res.

[94] Chae YK, Pan A, Davis AA (2016). Biomarkers for PD-1/PD-L1 blockade therapy in non-small-cell lung cancer: is PD-L1 expression a good marker for patient selection?. Clin Lung Cancer.

[95] Le DT, Uram JN, Wang H (2015). PD-1 Blockade in tumors with mismatch-repair deficiency. N Engl J Med.

[96] Havel JJ, Chowell D, Chan TA (2019). The evolving landscape of biomarkers for checkpoint inhibitor immunotherapy. Nat Rev Cancer.

[97] Dissaux G, Visvikis D, Da-Ano R, et al. Pre-treatment ^18^F-FDG PET/CT Radiomics predict local recurrence in patients treated with stereotactic radiotherapy for early-stage non-small cell lung cancer: a multicentric study. J Nucl Med. 2019;jnumed.119.228106. 10.2967/jnumed.119.228106 31732678

[98] Oikonomou A, Khalvati F, Tyrrell PN (2018). Radiomics analysis at PET/CT contributes to prognosis of recurrence and survival in lung cancer treated with stereotactic body radiotherapy. Sci Rep.

[99] Sampath S, Rahmanuddin S, Sahoo P (2019). Change in apparent diffusion coefficient is associated with local failure after stereotactic body radiation Therapy for Non-Small Cell Lung Cancer: a prospective clinical trial. Int J Radiat Oncol Biol Phys.

[100] Luo H, Ge H, Cui Y (2018). Systemic inflammation biomarkers predict survival in patients of early stage non-small cell lung cancer treated with stereotactic ablative radiotherapy - a single center experience. J Cancer.

[101] Yu Y, Zeng D, Ou Q (2019). Association of survival and immune-related biomarkers with immunotherapy in patients with non-small cell lung cancer: a meta-analysis and individual patient-level analysis. JAMA Netw Open.

[102] Louvel G, Bahleda R, Ammari S (2018). Immunotherapy and pulmonary toxicities: can concomitant immune-checkpoint inhibitors with radiotherapy increase the risk of radiation pneumonitis?. Eur Respir J.

[103] Manapov F, Roengvoraphoj O, Dantes M, Marschner S, Li M, Eze C (2018). Pneumonitis in Irradiated Lungs After Nivolumab: A brief communication and review of the literature. J Immunother.

[104] Shibaki R, Akamatsu H, Fujimoto M, Koh Y, Yamamoto N (2017). Nivolumab induced radiation recall pneumonitis after two years of radiotherapy. Ann Oncol.

[105] Tian S, Switchenko JM, Buchwald ZS, et al. Lung stereotactic body radiation therapy and concurrent immunotherapy: a multicenter safety and toxicity analysis. Int J Radiat Oncol Biol Phys. 2020;S0360-3016(19)34548-1. 10.1016/j.ijrobp.2019.12.030 PMC7747230 31982496

[106] Wang DY, Salem JE, Cohen JV (2018). Fatal toxic effects associated with immune checkpoint inhibitors: a systematic review and meta-analysis. JAMA Oncol.

[107] Hwang WL, Niemierko A, Hwang KL (2018). Clinical outcomes in patients with metastatic lung cancer treated with PD-1/PD-L1 inhibitors and thoracic radiotherapy. JAMA Oncol.

[108] Osorio JC, Ni A, Chaft JE (2017). Antibody-mediated thyroid dysfunction during T-cell checkpoint blockade in patients with non-small-cell lung cancer. Ann Oncol.

[109] Haratani K, Hayashi H, Chiba Y (2018). Association of immune-related adverse events with nivolumab efficacy in non-small-cell lung cancer. JAMA Oncol.

[110] Tamiya A, Tamiya M, Nakahama K (2017). Correlation of radiation pneumonitis history before nivolumab with inset of interstitial lung disease and progression-free survival of patients with pre-treated advanced non-small cell lung cancer. Anticancer Res.

[111] Horvat TZ, Adel NG, Dang TO (2015). Immune-related adverse events, need for systemic immunosuppression, and effects on survival and time to treatment failure in patients with melanoma treated with ipilimumab at Memorial Sloan Kettering Cancer Center. J Clin Oncol.

[112] De Felice KM, Gupta A, Rakshit S (2015). Ipilimumab-induced colitis in patients with metastatic melanoma. Melanoma Res.

[113] Puzanov I, Diab A, Abdallah K (2017). Managing toxicities associated with immune checkpoint inhibitors: consensus recommendations from the Society for Immunotherapy of Cancer (SITC) Toxicity Management Working Group. J Immunother Cancer.

